# “Roar” of *bla*_NDM-1_ and “silence” of *bla*_OXA-58_ co-exist in *Acinetobacter pittii*

**DOI:** 10.1038/srep08976

**Published:** 2015-03-10

**Authors:** Shuru Zhou, Xin Chen, Xiaobin Meng, Guoxiong Zhang, Jie Wang, Dongsheng Zhou, Xuemin Guo

**Affiliations:** 1 Institute of Human Virology, Zhongshan School of Medicine, Sun Yat-Sen University, Guangzhou, China; 2 Key Laboratory of Tropical Disease Control at Sun Yat-Sen University, Ministry of Education, Guangzhou, China; 3 Meizhou People's Hospital, Meizhou, China; 4 State Key Laboratory of Pathogen and Biosecurity, Beijing Institute of Microbiology and Epidemiology, Beijing, China; 5 Department of Biochemistry, Zhongshan School of Medicine, Sun Yat-Sen University, Guangzhou, China

## Abstract

*Acinetobacter pittii* 44551 was recovered from a patient with gout combined with tuberculosis and was found to harbor the carbapenemase genes *bla*_NDM-1_ and *bla*_OXA-58_ on two different plasmids pNDM-44551 and pOXA58-44551, respectively. pNDM-44551 displayed high self-transferability across multiple bacterial species, while pOXA58-44551 was likely co-transferable with pNDM-44551 into *A. baumannii* receipts. pNDM-44551 was a close variant of the previously characterized pNDM-BJ01, and the *bla*_NDM-1_ gene cluster was arranged sequentially as *orfA*, IS*Aba14*, *aphA6*, IS*Aba125*, *bla*_NDM-1_, *ble*_MBL_, *ΔtrpF*, *dsbC*, *tnpR*, and *zeta*. pOXA58-44551 was a repAci9-containing plasmid, and *bla*_OXA-58_ was embedded in a *372F*-IS*Aba3*-like-*bla*_OXA-58_-IS*Aba3* structure. The mobile genetic platforms of *bla*_NDM-1_ and *bla*_OXA-58_ herein showed some differences from their previously characterized variants. The production of NDM-1 in strain 44551 contributed the majority to its high resistance to carbapenems, while the *bla*_OXA-58_ stayed silent most likely due to the lack of an upstream promoter to drive its transcription. Increased surveillance of *Acinetobacter* co-harboring *bla*_NDM-1_ (active) and *bla*_OXA-58_ (either active or silent) is urgently needed.

A*cinetobacter* spp. are important opportunistic pathogens closely linked to nosocomial infections, and the most common species from clinical samples is *A. baumannii*, followed by *A. pittii* and *A. nosocomialis*^1^. Worldwide increasing emergence of carbapenem-resistant bacteria including *Acinetobacter* has caused concern over the limited availability of effective antimicrobial agents in the clinic.

The most prevalent mechanism of carbapenem resistance in *Acinetobacter* is the elevated expression of OXA-type carbapenemase genes, mainly including the horizontally acquired *bla*_OXA-23_-like, *bla*_OXA-24_-like and *bla*_OXA-58_-like genes as well as the intrinsic *bla*_OXA-51_-like genes^2^,^3^. The *bla*_OXA-58_-like and *bla*_OXA-24_-like genes are much less frequently detected relative to the *bla*_OXA-23_-like genes, but they can confer a high level of carbapenem resistance and cause local outbreaks^4^,^5^,^6^. The carbapenem hydrolytic activity of OXA-58 can be inhibited by NaCl in a Tyr^144^-Gly-Asn motif-dependent pattern, and this property has been used to screen for the expression of *bla*_OXA-58_^7^.

At least four types of metallo-β-lactam (MBL) carbapenemase, i.e., IMP, VIM, SIM, and NDM, have been identified in *Acinetobacter*, and these MBLs have lower detection rates but higher carbapenemase activities relative to OXAs^8^,^9^. Originally found in *Klebsiella pneumoniae* in 2008, *bla*_NDM_ has been identified globally in many bacteria and received great attention partly due to its high transferability across different bacterial species, its frequent co-occurrence with other resistance genes, and the global spread of *bla*_NDM_-carrying strains^10^,^11^,^12^,^13^,^14^,^15^. A large collection of *bla*_NDM_-containing plasmids from different bacterial species have been characterized (Table S1 and the references therein). The *bla*_NDM_ genetic surroundings are commonly composed of IS*Aba125* (intact or truncated) and *ble*_MBL_-*ΔtrpF*, which are upstream and downstream of *bla*_NDM_, respectively, suggesting a similar origin of these *bla*_NDM_ genes detected (Table S1). Most of the *bla*_NDM_ genetic platforms from *Acinetobacter* are a Tn*125*-related sequence, with a few exceptions containing the truncated forms of this genetic context (Table S1). Tn*125* is a *bla*_NDM_-containing composite transposon originally described by Pfiefer et al.^16^, and it has been proposed to be involved in the *bla*_NDM_ genes dissemination in *Acinetobacter*^17^. Almost all the well-characterized *bla*_NDM_-containing plasmids from *Acinetobacter* in China are closely related to the plasmid pNDM-BJ01^18^ by sharing a novel type IV secretion system backbone (Table S1 and references therein).

Coexistence of *bla*_NDM_ and *bla*_OXA_ has been described in *Acinetobacter*, e.g. *bla*_OXA-23_ and *bla*_NDM-1_ in *A. baumannii* from India^19^ and the Czech Republic^20^, and *bla*_NDM-1_, *bla*_OXA-23_ and *bla*_IMP_ in *A. baumannii* from China^21^. However, it remains unclear whether and how these co-existing carbapenemase genes are expressed to contribute to drug resistance. The present study describes the genetic environment, transferability, and antibiotic susceptibility of *bla*_NDM-1_ and *bla*_OXA-58_ harbored on different plasmids in a single clinical *A. pittii* isolate from China.

## Results and Discussion

### *bla*_NDM-1_ and *bla*_OXA-58_ on different plasmids in a single *A. pittii* isolate

A total of 31 carbapenem-resistant *Acinetobacter* isolates were collected at the Meizhou People's Hospital, Guangdong Province, China, from June 2010 to December 2012. They were classified into *A. pittii* (n = 1, designated 44551), *A*. *nosocomialis* (n = 2), and *A. baumannii* (n = 28). PCR screening for the MBL and OXA carbapenemase genes in these strains indicated the presence of *bla*_NDM-1_ and *bla*_OXA-58_ in strain 44551 and the habitation of *bla*_OXA-23_ in 21 *A. baumannii* isolates (67.7%), while all the other PCR reactions gave negative results. DNA sequencing further confirmed the presence of *bla*_NDM-1_ and *bla*_OXA-58_ in 44551. PCR detection of the extended spectrum β-lactamase genes *bla*_TEM_, *bla*_CTX-M_, *bla*_PER_, *bla*_SHV_, *bla*_DHA_, and *bla*_CMY_^22^,^23^,^24^,^25^ in 44551 showed negative results. The observation of *bla*_OXA-23_ as the most prevalent carbapenemase gene in *A. baumannii* is consistent with previous findings^26^.

*A. pittii* 44551 was isolated from the sputum of a 62-year-old male hospitalized for gouty arthritis with a skin soft tissue infection combined with pulmonary tuberculosis (TB) in August 2011. After the initiation of anti-tuberculosis treatment with HREZ (isoniazid, rifampicin, ethambutol hydrochloride, and pyrazinamide), the patient's sputum samples were screened weekly for *Mycobacterium tuberculosis*. The patient's pulmonary infection symptoms, including fever, weakness and coughing, were relieved in response to anti-TB therapy, and his sputum acid-fast stain gave negative results after two weeks of treatment. After one month of hospitalization, the patient improved perceptibly with stable vital signs. He was discharged with instructions to continue taking 3HREZ/9-12HRZ as prescribed and to undergo regular follow-up. Unfortunately, the patient did not return for regular checkup and has been out of touch with our clinic.

**Figure 1:**
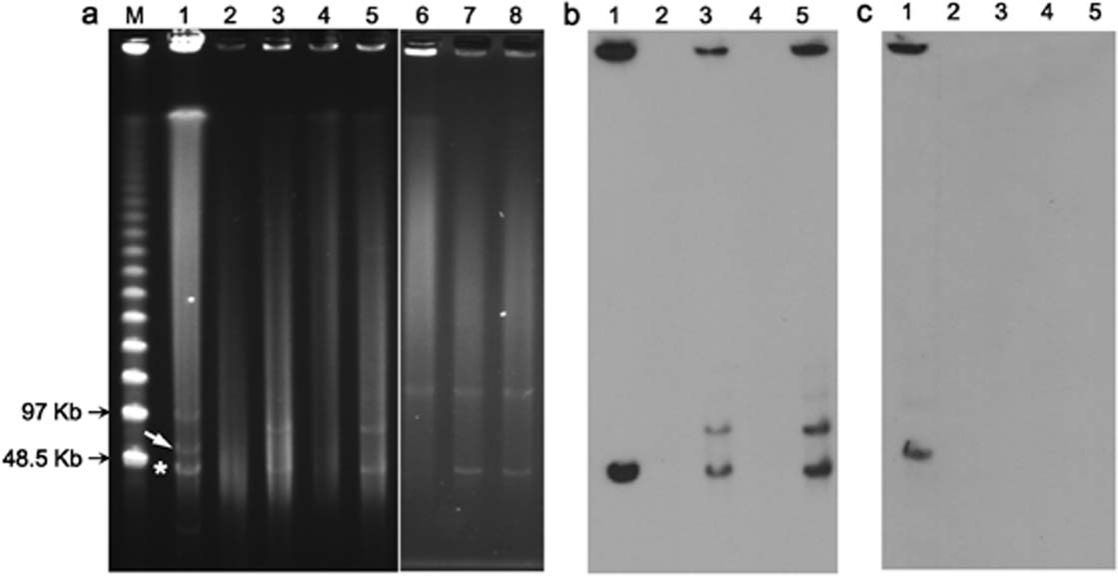
S1-PFGE and Southern blot assay of strain 44551 and its conjugants. Shown are EtBr-stained PFGE gel of S1-digested of genomic DNA samples (a), and subsequent Southern blot hybridization with the DIG-labeled probe specific to *bla*_NDM-1_ (b) or *bla*_OXA-58_ (c). The white arrow and star indicate the locations of *bla*_OXA-58_- and *bla*_NDM-1_-positive signals, respectively. Lane 1: 44551; 2: J53; 3: J53-44551; 4: EC600; 5: EC600-44551; 6: MZPB; 7: MZPB-44551; 8: MZPB-44551^OXA58+^; M: marker.

S1-nuclease pulsed-field gel electrophoresis (S1-PFGE) assay (Fig. 1a) showed that *A. pittii* 44551 harbored four different plasmids at ~20 kb, ~40 kb, ~50 kb and ~90 kb. Subsequent Southern hybridization (Fig. 1b) with a probe specific to *bla*_NDM-1_ or *bla*_OXA-58_ revealed that these two genes were located on the ~40 kb (designated pNDM-44551) and ~50 kb (pOXA58-44551) plasmids, respectively. Using the PCR-based replicon typing scheme of *A. baumannii*^27^, repAci9 was detected in pOXA58-44551 but no classified type of replicase could be identified from pNDM-44551.

Mating out experiments showed that plasmid pNDM-44551 could be transferred from 44551 into *E. coli* J53 and EC600, and *A. baumannii* MZPB at similar frequencies of 2.36 × 10^−2^, 2.81 × 10^−2^, and 3.54 × 10^−2^ per donor cell, respectively. The initial cultures of all the chosen J53 and EC600 conjugant clones and most MZPB clones were *bla*_NDM-1_-positive and *bla*_OXA-58_-negative as revealed by PCR/sequencing. A single resulting conjugant from each of these J53, EC600, and MZPB conjugants, designated J53-44551 EC600-44551, and MZPB-44551, respectively, was selected for S1-PFGE/Southern hybridization (Fig. 1a and 1b). Besides detection of ~40 kb pNDM-44551 in all the tested conjugants, an additional *bla*_NDM-1_-positive signal at ~80 kb was found in J53-44551 and EC600-44551, suggesting that a recombination event occurred for pNDM-44551 upon conjugal transfer from *Acinetobacter* to *Enterobacteriaceae*. The above observations denoted high self-transferability of pNDM-44551 across multiple bacterial species.

A weak PCR signal of *bla*_OXA-58_ was observed in a small portion of the *bla*_NDM-1_-positive MZPB-44551 clones at a 1:10 ratio, as well as in their passage cultures, which was further confirmed by sequencing. Meanwhile, Random Amplified Polymorphic DNA showed that these *bla*_OXA-58_- and *bla*_NDM-1_-positive conjugants were MZPB-originated species. The PCR results of one of the above conjugants, designated MZPB-44551^OXA58+^, were shown in Fig. S1a and S1b. Nonetheless, the pOXA58-44551 signal was invisible by S1-PFGE/Southern hybridization in MZPB-44551^OXA58+^ (Fig. 1a), which was attributable to the very low copy number of pOXA58-44551. In addition, no PCR signal of *bla*_OXA-58_ was detected in all the *bla*_NDM-1_-haboring J53 or EC600 conjugants tested, which is consistent with the fact that *bla*_OXA-58_ has only been found in *Acinetobacter*^28^. In all, it seemed that pOXA58-44551 could co-transfer with pNDM-44551 only into the *Acinetobacter* recipient.

Plasmid pBBRIMCS3-NDM-1, containing the IS*Aba125*-*bla*_NDM-1_ fragment cloned from pNDM-44551, was transformed into *E. coli* DH5α, and the resulting transformant clone was named DH5α-NDM1. All clones of 44551, DH5α-NDM1, J53-44551, and EC600-44551 showed high resistance to the penicillin/cephalosporin drugs tested. 44551 and DH5α-NDM1 also displayed high resistance to IPM and MEM, but J53-44551 and EC600-44551 remained susceptible to these two carbapenem drugs (Table 1). Therefore, we speculated that more functional NDM-1 proteins might be present in 44551 and DH5α-NDM1 than in J53-44551 and EC600-44551, although there was no discernible difference in the pNDM-44551 copy number among these strains (Fig. 1a and 1b). This speculation was supported by the northern blot and quantitative reverse-transcription PCR (RT-qPCR) assays, which showed that the *bla*_NDM-1_ mRNA abundance in 44551 or DH5α-NDM1 was much higher than that in J53-44551 or EC600-44551 (Fig. S2).

It is worth noting that MZPB-44551 showed distinct antimicrobial susceptibility profile from 44551 across almost all the tested β-lactams (Table 1), while the pNDM-44551 copy number (Fig. 1a) or the *bla*_NDM-1_ mRNA abundance (Fig. S2) was comparable between these two strains. Based on the spontaneous PB-resistance of MZPB under PB selection, it is reasonable to speculate that this distinction may be mainly due to different species backgrounds.

### Genetic surroundings of *bla*_NDM-1_

pNDM-BJ01 was recovered from *A. lwoffii* in China in 2012 and harbored four regions encoding for conjugate transfer, plasmid replication and stability, a type IV secretion system, and the *bla*_NDM-1_ gene cluster^18^. Primer walking combined PCR/sequencing indicated that pNDM-44551 contained 41 of the 46 CDSs annotated in pNDM-BJ01 with >99% sequence identity and the missing five CDSs were located in a tandem manner within the *bla*_NDM-1_ gene cluster.

**Figure 2:**
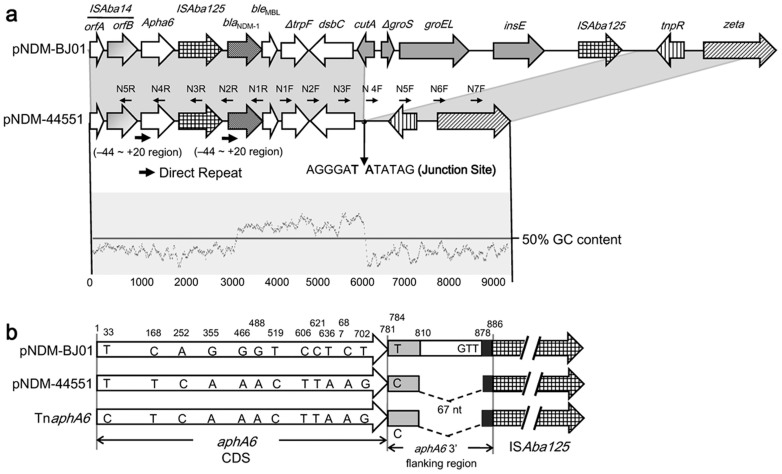
Schematic alignment of *bla*_NDM-1_ genetic surroundings. Genes and their transcriptional orientations are represented by differently patterned horizontal arrows. *orfA* and *orfB*: *ISAba*14 transposase; *aphA6*: aminoglycoside 3′-phosphotransferase; *bla*_NDM-1_: New Delhi metallo-β-lactamase 1; *ble*_MBL_: bleomycin resistance protein; *ΔtrpF*: truncated phosphoribosylanthranilate isomerase; *dsbC*: tat twin-arginine translocation pathway signal sequence domain protein; *cutA*: periplasmic divalent cation tolerance protein; *ΔgroS* and *groEL*: chaperonin subunits; ins*E*: IS*CR3* transposase. (a) The *bla*_NDM-1_ gene clusters from pNDM-44551 and pNDM-BJ01. The identical DNA regions are shaded in gray, the GC skew is shown with a sliding window of 100 nucleotides, and the primers used for PCR/sequencing are marked with thin black arrows as well as corresponding primer names. (b) Schematic showing the sequence differences of *aphA6* CDSs and their 3′ flanking regions from pNDM-BJ01, pNDM-44551, and Tn*aph6*.

The *bla*_NDM-1_ gene cluster of pNDM-44551 was arranged sequentially as *orfA* and *orfB* of IS*Aba14*, *aphA6*, IS*Aba125*, *bla*_NDM-1_, *ble*_MBL_, *ΔtrpF*, *dsbC*, *tnpR*, and *zeta* from left to right; a five-gene region (*cutA*, *ΔgroS*, *groEL*, *insE*, and IS*Aba125*) located between *dsbc* and *tnpR* of pNDM-BJ01 was absent from pNDM-44551; the cleavage of the above DNA region led to a 97 bp 3′-remnant (GC content ≈ 60%) of the *cutA* coding region (CDS) to be spliced directly with a 372 bp 3′-flanking sequence (GC content ≈ 40%) of *tnpR* at the junction site AGGGAT-ATATAG, generating a ‘new' *dsbC*-*tnpR* intergenic region in pNDM-44551 (Fig. 2a).

The *aphA6* gene was usually found adjacently upstream of the IS*Aba125*-*bla*_NDM-1_ structure in the pNDM-BJ01-like plasmids (Table S1). It has been postulated that a Tn*125* transposon structure inserts into the non-coding region downstream of *aphA6*, which is evidenced by the 3-bp GTT target site duplication at the point of insertion as shown in pNDM-BJ01^18^. A 64 bp direct repeat was observed flanking the IS*Aba125*-*bla*_NDM-1_ insertion in pNDM-44551, and each monomer was composed of the first 20 bp of the CDS of *aphA6* or *bla*_NDM-1_ together with its upstream 44 bp sequence (Fig. 2a), which was consistent with the previous report that *bla*_NDM-1_ is a chimeric gene resulted from the in-frame fusion of a preexisting *bla*_NDM-1_ with *aphA6*^29^.

A total of 11 single nucleotide polymorphism (SNP) sites were present in the *aphA6* CDS of pNDM-44551 relative to pNDM-BJ01, resulting in three amino acid (a.a.) changes of L84F, A156T, and R163K. In addition, a 67 bp deletion occurred within the 3′ flanking region of *aphA6* of pNDM-44551 compared with pNDM-BJ01 (Fig. 2b). The *aphA6* CDS together with its 3′ flanking region of pNDM-44551 was almost identical to the counterpart of Tn*aphA6* (accession number JF343537, located in the chromosome of *A. baumannii* from Australia) and that of pWH8144 (accession number JG241792, in *A. baumannii* from China), with only one SNP mismatch (T to C) at the 5′ end of *aphA6*. By contrast, this kind of *aphA6* gene considerably differed from the counterpart of pNDM-BJ01 (Fig. 2b). *aphA6* has been proposed to be an ancestral gene in *Acinetobacter* and possesses considerable nucleotide polymorphism^30^. There might be two possible explanations for the aforesaid *aphA6* sequence difference: 1) the original recombination of *aphA6* with *bla*_NDM-1_ may be two independent events in pNDM-44551 and pNDM-BJ01, and 2) homologous recombination may occur to induce an *aphA6* swap upon the spread of the *aphA6*-IS*Aba125*-*bla*_NDM-1_-containing plasmid into an *aphA6*-carrying *Acinetobacter* strain.

The above observations strongly suggest that pNDM-44551 represents a close derivate of pNDM-BJ01 although pNDM-44551 might have undergone multiple evolutionary events especially within the *bla*_NDM-1_ gene cluster. An array of the pNDM-44551 variants have been characterized in *Acinetobacter* from the mainland of China, such as pXBC1 in *A. johnsonii*^31^, pNDM-AB in *A. baumannii*^32^, and pAP-D499 in *A. pittii*^33^. The major genetic differences in the above plasmids were confined in the surrounding regions of *bla*_NDM-1_ (Table S1). It should be noted that the IS*Aba125* element upstream of *bla*_NDM-1_ is usually intact in *Acinetobacter* but often truncated in *Enterobacteriaceae* (Table S1), suggesting the probable spread of the *bla*_NDM-1_ genetic platforms from *Acinetobacter* to *Enterobacteriaceae*^13^,^18^,^29^,^34^

### Genetic surroundings of silent *bla*_OXA-58_

**Figure 3:**
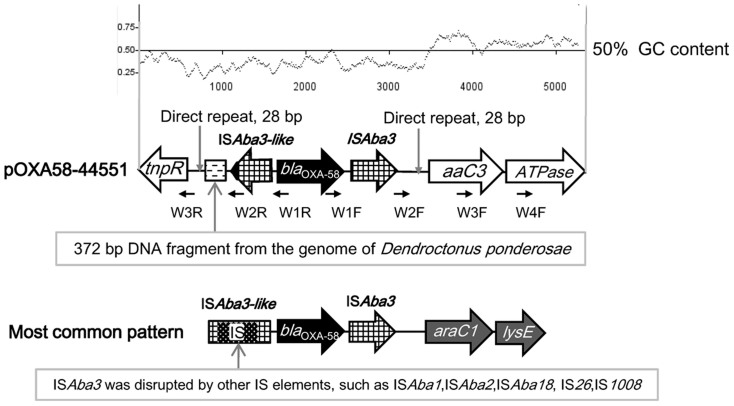
Schematic alignment of *bla*_OXA-58_ genetic surroundings. Shown are the *bla*_OXA-58_ gene cluster from pOXA58-44551 (upper panel), and that of the most common pattern (lower panel) characterized previously^35^,^36^,^37^,^38^,^39^,^40^. Genes and their transcriptional orientations are represented by horizontal arrows. The primers used for PCR/sequencing are marked with thin black arrows as well as corresponding primer names.

In general, *bla*_OXA-58_ is embedded in a conserved platform IS*Aba3*-like-*bla*_OXA-58_-IS*Aba3* in *Acinetobacter*, and the upstream IS*Aba3*-like element is often interrupted by other insertion sequence (IS) elements, which in turn provide the promoters enhancing the *bla*_OXA-58_ expression to mediate higher degrees of drug resistance compared with the parent intact IS*Aba3*-like^35^,^36^,^37^,^38^,^39^. Sequence analysis revealed that the *bla*_OXA-58_ gene of pOXA58-44551 was located between a downstream IS*Aba3* and an upstream IS*Aba3*-like, both of which were intact; the downstream IS*Aba3* was followed by a gene cluster *aaC3* (aminoglycoside N3′-acetyltransferase III)-*ATPase* (ATPase protein) (Fig. 3), which differed from the previously reported common pattern *araC1* (transcription regulator)-*lysE* (threonine efflux protein) in *bla*_OXA-58_ genetic contexts in *Acinetobacter* spp. from China^40^ and other countries^39^,^41^. The C-terminal 23 a.a. sequence of the transposase of the upstream IS*Aba3*-like element in pOXA58-44551 was replaced with an unknown 26 a.a. fragment, and moreover the 3′-untranslated region (3′-UTR) and the right inverted repeat (IRR) were lost (Fig. 3). This entire upstream IS*Aba3*-like element was essentially the same as the counterpart in the *bla*_OXA-58_ genetic structure in an *A. pittii* isolate AP04 from China^40^ and some unpublished *bla*_OXA-58_-containing sequences (accession numbers JX101647, FJ195389 and FJ200187), as well as in a *bla*_OXA-97_ genetic structure^36^. In addition, a 372 bp DNA fragment (named *372F*) as the left neighbor of the upstream IS*Aba3*-like element in pOXA58-44551 (Fig. 3) showed 100% sequence identity to that in the genome of the insect *Dendroctonus ponderosae* (APGK01007886.1). In all, *bla*_OXA-58_ was mobilized into the genetic platform *372F*-IS*Aba3*-like-*bla*_OXA-58_-IS*Aba3* (flanked by the 28 bp direct repeat; having a GC content much lower than those of the surrounding regions), which was further inserted into the backbone of pOXA58-44551 most likely through IS-mediated transposition.

**Figure 4:**
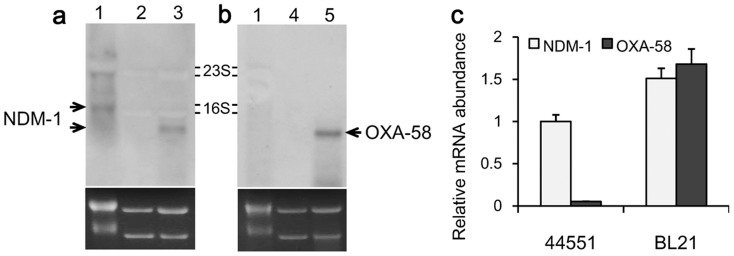
Detection of *bla*_NDM-1_ and *bla*_OXA-58_ transcripts. Total RNAs were extracted from strains 44551, BL21-NDM and BL21-OXA58. RNA samples were analyzed on 1.2% agarose gel followed by EtBr staining, and then subjected to Northern blot hybridization with the DIG-labeled probe specific to *bla*_NDM-1_ (a) and *bla*_OXA-58_ (b). IPTG was added in the cell cultures to induce the expression of *bla*_NDM-1_ or *bla*_OXA-58_. Lane 1: 44551; 2: BL21-NDM in the absence of IPTG; 3: BL21-NDM in the presence of IPTG; 4: BL21-OXA58 in the absence of IPTG; 5: BL21-OXA58 in the presence of IPTG. The calculated size of *bla*_NDM-1_ or *bla*_OXA-58_ transcript is about 1 kb. The EtBr staining of the 23S and 16S rRNA genes (2.9 kb and 1.5 kb, respectively) was used as loading control (lower panels). The relative mRNA abundances of *bla*_NDM-1_ or *bla*_OXA-58_ in 44551, BL21-NDM and BL21-OXA58 induced with IPTG, respectively, were detected by RT-qPCR (c). The 16S rRNA genes of 44551 and BL21 were employed as the internal control. The normalized mRNA abundance of *bla*_NDM-1_ in 44551 was set as 1. Results are expressed as mean ± SD.

The evidences described below from different aspects denoted that *bla*_OXA-58_ was expressed either very weakly or not at all in 44551. First, the addition of EDTA (the inhibitor of MBLs including NDM1) in the agar plates for bacterial cultivation made 44551 lose almost the entire resistance to the tested β-lactams, but the addition of excess NaCl (the inhibitor of OXA-58) had no effect on the resistance profile of 44551 (Table 1), indicating that the production of NDM-1 enzyme contributed the majority to β-lactams resistance while *bla*_OXA-58_ contributed little to the resistance. Second, the IS*Aba3*-like element upstream of *bla*_OXA-58_ in pOXA58-44551 was intact, suggesting a lack of the *bla*_OXA-58_-driven promoter which was usually provided by other inserted IS elements in the typical OXA-58-encoding genetic structures^35^,^36^,^37^,^38^,^39^ (Fig. 3). This postulation was further supported by a recent surveillance study, which showed that as many as 25 of the total of 32 *bla*_OXA-58_-positive *Acinetobacter* isolates recovered from 23 Chinese provinces remained susceptible to carbapenems, and all of them contained an intact IS*Aba3*-like element upstream of *bla*_OXA-58_, while the remaining non-susceptible isolates showed an insertion of additional IS element into the upstream IS*Aba3*-like element^42^. Third, repeated attempts to transform the plasmid pBBRIMCS3-OXA-58 (containing the IS*Aba3*-like-*bla*_OXA-58_ fragment cloned from pOXA58-44551) into *E. coli* DH5α failed to generate an ampicilin-resistant, *bla*_OXA-58_-haboring clone. Finally, northern blot with a probe specific to *bla*_OXA-58_ showed very weak or almost invisible signals in *A. pittii* 44551, but a very strong band could be detected in the BL21-OXA58 strain, which was an *E. coli* BL21 strain carrying the plasmid pET-28a-*bla*_OXA58_ (Fig. 4a and 4b). Consistent with the northern blot results, RT-qPCR assay revealed that the relative mRNA abundance of *bla*_OXA-58_ in strain 44551 was about 30-fold lower than that in BL21-OXA58, with the 16S rRNA genes being the internal control (Fig. 4c).

### Concluding remarks

In the present study, *bla*_NDM-1_ and *bla*_OXA-58_ were found to be harbored on two different plasmids in a single clinical *A*. *pittii* isolate named 44551, with the former gene expressing well and the latter one silent. Each of these two resistance genes was embedded in a genetic structure differing partly from its previously characterized variants. The *bla*_NDM-1_-carrying plasmid in 44551, conferring a high level of carbapenem resistance, showed a strong ability of horizontal transfer into *Acinetobacter* and *Enterobacteriaceae*. The silent *bla*_OXA-58_ has the potential to evolve into the active form due to additional IS element insertion driven by environmental pressures such as the presence of carbapenems. The fact that *A. pittii* 44551 colonized in the lower respiratory tract of the indicated hospitalized patient increased the possibility of the strain 44551 dissemination into hospital settings. Since the *Acinetobacter* strains co-harboring *bla*_NDM-1_ (active) and *bla*_OXA-58_ (either active or silent) have the potential to widely spread in China^42^, increased surveillance of these kinds of bacteria in hospital and community settings is urgently needed.

## Methods

### Clinical Acinetobacter isolates

Each clinical sample was inoculated onto MacConkey agar plates and the dominant strain was recovered and identified using Vitek II (BioMérieux, Durham). Discrimination of *Acinetobacter* was performed by one-tube multiplex PCR specific for *A. baumannii* identification^43^ and by 16S-23S rRNA intergenic spacer sequencing for other types of *Acinetobacter*^44^. All the primers used in this study are listed in Table S2.

### Detection of carbapenemase genes and their genetic contexts

The MBL genes *bla*_IMP_, *bla*_VIM_, *bla*_SIM_, and *bla*_NDM_ were screened by multiplex PCR^45^. The OXA genes *bla*_OXA-23_, *bla*_OXA-24_, and *bla*_OXA-58_ were detected individually by PCR. The plasmid sample was prepared from 44551 using a BAC/PAC DNA Isolation Maxi Kit (Omega Bio-Tek), and the flanking regions were sequenced by primer walking from both ends of *bla*_NDM-1_ or *bla*_OXA-58_. All PCR amplicons were subjected to DNA sequencing with an ABI 3700 sequencer.

### Plasmid construction and electrotransformation

The *bla*_NDM-1_ or *bla*_OXA-58_ coding region together with its immediately upstream IS element was amplified from 44551 and then cloned into the cloning vector pBBRIMCS3^46^, generating the recombinant plasmid pBBRIMCS3-NDM-1 or pBBRIMCS3-OXA-58, respectively. The entire coding region of *bla*_OXA-58_ was cloned into the expression vector pET-28a, generating pET-28a-*bla*_OXA-58_. pBBRIMCS3-NDM-1 or pBBRIMCS3-OXA-58 was transformed into *E. coli* DH5α through electrotransformation, while pET-28a-*bla*_OXA-58_ was transformed into *E. coli* BL21, with an attempt to obtain the *E. coli* clone expressing the corresponding carbapenemase enzyme.

### Conjugal transfer

*A. pittii* 44551 harboring *bla*_NDM-1_ and *bla*_OXA-58_ was used as the donor, and *E. coli* J53 (NaN_3_ resistant), EC600 (rifampin resistant), and *A. baumannii* MZPB were used as the recipients. MZPB is a homemade polymyxin-resistant derivate of a clinical isolate *A. baumannii* MZ and remains susceptible to β-lactams. Membrane mating experiments were performed on Mueller-Hinton (MH) agar^47^. After 18 h of incubation, the mixed cultures were suspended in MH broth, and plated onto MH agar containing ampicillin (50 μg/ml) and NaN_3_ (200 μg/ml) for J53, ampicillin (50 μg/ml) and rifampin (500 μg/ml) for EC600, and ampicillin (50 μg/ml) and polymyxin (20 μg/ml) for MZPB. Conjugants were picked randomly from the original selective plates and inoculated into the selective LB broth, these initial cultures were used for PCR-based screening for the presence of *bla*_NDM-1_ and *bla*_OXA-58_. To exclude the donor strain contamination, the initial culture of positive conjugants were spread onto the Amp^+^/PB^+^ plate, then the second-passage colonies were randomly picked for each strain and subjected for PCR detection of *bla*_NDM-1_ and *bla*_OXA-58_. The strain species were differentiated by Random Amplified Polymorphic DNA with two short primers M13 and AP2^48^.

### Antimicrobial susceptibility testing

The MIC values for each indicated strain cultured on the MH plates were measured using Etest (AB bioMérieux, Solna, Sweden). The results were interpreted according to the Clinical and Laboratory Standards Institute (CLSI) guidelines and the British Society for Antimicrobial Chemotherapy (SAC) breakpoints^49^,^50^.

### S1-PFGE and Southern blot

Bacterial genomic DNA was prepared in agarose plugs and digested with S1 nuclease (Takara). The linearized plasmids and partially digested genomic DNA were separated through the CHEF-Mapper XA PFGE system (Bio-Rad). The DNA fragments were stained with ethidium bromide (EtBr), transferred to a Hybond N+ membrane (GE Amersham Biosciences) and hybridized with the DIG-labeled probe specific to *bla*_NDM-1_ or *bla*_OXA-58_. Probe labeling and signal detection were carried out with DIG high primer DNA labeling and detection starter kit II according to the manufacturer's instructions (Roche Diagnostics).

### RNA extraction and Northern blot

By using Trizol Reagent (Life Technologies), total RNA was isolated from the overnight culture of each indicated strain with or without addition of 1 mM IPTG. The RNA samples were analyzed on a formaldehyde-containing 1.2% agarose gel. Subsequent EtBr staining, membrane transfer, probe labeling and hybridization, and signal detection were carried out as above.

### RT-qPCR

5 μg of RNA treated with DNase I (Promega) was subjected to reverse transcription by using random hexamers and PrimeScript RT reagents (Takara). Simultaneously, a control reverse transcription reaction without reverse transcriptase was performed to rule out genomic DNA contamination. The cDNA reactions were diluted 1:5 in water as the template for PCR detection of *bla*_NDM-1_ or *bla*_OXA-58_, and diluted 1:500 for amplification of the 16S rRNA genes. Each PCR reaction contained 2 μl of cDNA, 8 μl of forward and reverse primers (each at 0.75 μM), and 10 μl of SYBR green PCR Supermix (Bio-Rad). Three independent bacterial cultures (total RNA samples) were employed as biological replicates, and each RNA sample was analyzed in triplicate in PCR. The PCR parameters were 95°C for 10 min, followed by 40 cycles of 95°C for 5 s and 60°C for 30 s, using Bio-Rad CFX96 thermocycler. The detecting mRNA levels of *bla*_NDM-1_ or *bla*_OXA-58_ were normalized to those of the 16S rRNA genes.

### Plasmid replicon typing

Plasmid pOXA58-44551 was recovered from the S1-PFGE gel and used as the template for PCR detection of replicase genes using the *A. baumannii* PCR-based replicon typing scheme^27^. The variants belonging to the same group of replicases were recognized by further PCR amplicon sequencing.

### Nucleotide sequence accession numbers

The *bla*_NDM-1_ or *bla*_OXA-58_ gene cluster reported herein was deposited in GenBank with the accession number KF208467 or KF208466, respectively.

## Supplementary Material

Supplementary Information Supplementary data

## Figures and Tables

**Table 1:** Antimicrobial susceptibility profiles

Strain	MIC (μg/ml)														
	AB	TZP	SCF	CAZ	CTX	FEP	IPM	MEM	ATM	GM	AMK	CIP	PMB	MIN	TGC
44551	24	>256	24	>256	>256	>256	>32	>32	4	24	64	>32	0.50	0.023	0.125
J53-44551	>256	32	64	>256	32	1.0	1.5	1	0.23	0.125	1	0.008	0.5	0.75	0.064
J53	2	0.125	0.032	0.125	0.125	0.047	0.38	0.064	0.047	0.125	1	0.008	0.38	0.75	0.064
EC600-44551	>256	64	48	>256	96	4	3	2	0.047	0.25	2	0.094	0.38	1.0	0.047
EC600	2	1.5	0.094	0.25	0.125	0.064	0.38	0.023	0.047	0.25	2	0.125	0.19	0.75	0.047
MZPB-44551	1	2	0.094	128	96	4	2	2	4	0.125	1	0.032	20	0.023	0.064
MZPB	1	2	0.094	2	2	1	0.094	0.094	4	0.125	1	0.032	20	0.023	0.064
DH5α-NDM1	>256	>256	>256	>256	>256	>32	16	12	0.047	0.125	1	0.008	0.38	0.75	0.064
DH5α	2	0.125	0.032	0.25	0.125	0.064	0.38	0.064	0.047	0.125	1	0.008	0.38	0.75	0.064
44551 (100 μM EDTA)	0.5	0.5	0.125	1	0.38	0.5	0.38	0.38	4	24	64	>32	0.5	0.023	0.125
44551 (200 mM NaCl)	16	64	16	>256	>256	>256	>32	>32	4	24	64	>32	0.5	0.023	0.125

AB, penicillin/sulbactam; TZP, piperacillin/tazobactam; SCF, cefoperazone/sulbactam 2:1; CAZ, ceftazidime; CTX, cefotaxime; FEP, cefepime; IPM, imipenem; MEM, meropenem; ATM, aztreonam; GEN, gentamicin; AMK, amikacin; CIP, ciprofloxacin; PMB, polymyxin B; MIN, minocycine; TGC, tigecycline. 44551 (100 μM EDTA), and 44551 (200 mM NaCl): cultivation of 44551 on the MH plates containing 100 μM EDTA, and 200 mM NaCl, respectively.

## References

[b1] Nemec A. *et al.* Genotypic and phenotypic characterization of the *Acinetobacter calcoaceticus-Acinetobacter baumannii* complex with the proposal of *Acinetobacter pittii* sp. nov. (formerly *Acinetobacter* genomic species 3) and *Acinetobacter nosocomialis* sp. nov. (formerly *Acinetobacter* genomic species 13TU). Res. Microbiol. 162, 393–404 (2011). 21320596 10.1016/j.resmic.2011.02.006

[b2] Abbott I., Cerqueira G. M., Bhuiyan S. & Peleg A. Y. Carbapenem resistance in *Acinetobacter baumannii*: laboratory challenges, mechanistic insights and therapeutic strategies. Expert Rev. Anti. Infect. Ther. 11, 395–409 (2013). 23566149 10.1586/eri.13.21

[b3] Poirel L. & Nordmann P. Carbapenem resistance in *Acinetobacter baumannii*: mechanisms and epidemiology. Clin. Microbiol. Infect. 12, 826–836 (2006). 16882287 10.1111/j.1469-0691.2006.01456.x

[b4] Mendes R. E., Bell J. M., Turnidge J. D., Castanheira M. & Jones R. N. Emergence and widespread dissemination of OXA-23, -24/40 and -58 carbapenemases among *Acinetobacter* spp. in Asia-Pacific nations: report from the SENTRY Surveillance Program. J. Antimicrob. Chemother. 63, 55–59 (2009). 18957398 10.1093/jac/dkn434

[b5] Moro M. *et al.* An outbreak caused by multidrug-resistant OXA-58-positive *Acinetobacter baumannii* in an intensive care unit in Italy. J. Hosp. Infect. 68, 97–99 (2008). 18063199 10.1016/j.jhin.2007.10.007

[b6] Castanheira M., Wanger A., Kruzel M., Deshpande L. M. & Jones R. N. Emergence and clonal dissemination of OXA-24- and OXA-58-producing *Acinetobacter baumannii* strains in Houston, Texas: report from the SENTRY Antimicrobial Surveillance Program. J. Clin. Microbiol. 46, 3179–3180 (2008). 18768660 10.1128/JCM.00988-08 PMC2546771

[b7] Poirel L. *et al.* OXA-58, a novel class D β-lactamase involved in resistance to carbapenems in *Acinetobacter baumannii*. Antimicrob. Agents Chemother. 49, 202–208 (2005). 15616297 10.1128/AAC.49.1.202-208.2005 PMC538857

[b8] Cornaglia G., Giamarellou H. & Rossolini G. M. Metallo-beta-lactamases: a last frontier for beta-lactams? Lancet Infect. Dis. 11, 381–393 (2011). 21530894 10.1016/S1473-3099(11)70056-1

[b9] Gupta V. Metallo beta lactamases in *Pseudomonas aeruginosa* and *Acinetobacter* species. Expert Opin. Investig. Drugs 17, 131–143 (2008). 10.1517/13543784.17.2.131 18230049

[b10] Bushnell G., Mitrani-Gold F. & Mundy L. M. Emergence of New Delhi metallo-beta-lactamase type 1-producing enterobacteriaceae and non-enterobacteriaceae: global case detection and bacterial surveillance. Int. J. Infect. Dis. 17, e325–333, 10.1016/j.ijid.2012.11.025 (2013). 23332300

[b11] Yong D. *et al.* Characterization of a new metallo-beta-lactamase gene, *bla*_NDM-1_, and a novel erythromycin esterase gene carried on a unique genetic structure in *Klebsiella pneumoniae* sequence type 14 from India. Antimicrob. Agents Chemother. 53, 5046–5054 (2009). 19770275 10.1128/AAC.00774-09 PMC2786356

[b12] Villa L., Poirel L., Nordmann P., Carta C. & Carattoli A. Complete sequencing of an IncH plasmid carrying the *bla*_NDM-1_, *bla*_CTX-M-15_ and *qnrB1* genes. J. Antimicrob. Chemother. 67, 1645–1650 (2012). 22511638 10.1093/jac/dks114

[b13] Poirel L., Dortet L., Bernabeu S. & Nordmann P. Genetic features of *bla*_NDM-1_-positive *Enterobacteriaceae*. Antimicrob. Agents Chemother. 55, 5403–5407 (2011). 21859933 10.1128/AAC.00585-11 PMC3195013

[b14] Wailan A. M. & Paterson D. L. The spread and acquisition of NDM-1: a multifactorial problem. Expert Rev. Anti. Infect. Ther. 12, 91–115 (2014). 24308710 10.1586/14787210.2014.856756

[b15] Dortet L., Poirel L. & Nordmann P. Worldwide dissemination of the NDM-type carbapenemases in Gram-negative bacteria. Biomed. Res. Int. 2014, 249856, 10.1155/2014/249856 (2014). 24790993 PMC3984790

[b16] Pfeifer Y. *et al.* Molecular characterization of *bla*_NDM-1_ in an *Acinetobacter baumannii* strain isolated in Germany in 2007. J. Antimicrob. Chemother. 66, 1998–2001 (2011). 21693460 10.1093/jac/dkr256

[b17] Poirel L. *et al.* Tn*125*-related acquisition of *bla*_NDM_-like genes in *Acinetobacter baumannii*. Antimicrob. Agents Chemother. 56, 1087–1089 (2012). 22143526 10.1128/AAC.05620-11 PMC3264265

[b18] Hu H. *et al.* Novel plasmid and its variant harboring both a *bla*_NDM-1_ gene and type IV secretion system in clinical isolates of *Acinetobacter lwoffii*. Antimicrob. Agents Chemother. 56, 1698–1702 (2012). 22290961 10.1128/AAC.06199-11 PMC3318331

[b19] Karthikeyan K., Thirunarayan M. A. & Krishnan P. Coexistence of *bla*_OXA-23_ with *bla*_NDM-1_ and *armA* in clinical isolates of *Acinetobacter baumannii* from India. J. Antimicrob. Chemother. 65, 2253–2254 (2010). 20650909 10.1093/jac/dkq273

[b20] Krizova L., Bonnin R. A., Nordmann P., Nemec A. & Poirel L. Characterization of a multidrug-resistant *Acinetobacter baumannii* strain carrying the *bla*_NDM-1_ and *bla*_OXA-23_ carbapenemase genes from the Czech Republic. J. Antimicrob. Chemother. 67, 1550–1552 (2012). 22366189 10.1093/jac/dks064

[b21] Chen Z. *et al.* Coexistence of *bla*_NDM-1_ with the prevalent *bla*_OXA23_ and *bla*_IMP_ in pan-drug resistant *Acinetobacter baumannii* isolates in China. Clin. Infect. Dis. 52, 692–693 (2011). 21292674 10.1093/cid/ciq231

[b22] Feizabadi M. M. *et al.* Distribution of *bla*_TEM_, *bla*_SHV_, *bla*_CTX-M_ genes among clinical isolates of *Klebsiella pneumoniae* at Labbafinejad Hospital, Tehran, Iran. Microb. Drug Resist. 16, 49–53 (2010). 19961397 10.1089/mdr.2009.0096

[b23] Perez-Perez F. J. & Hanson N. D. Detection of plasmid-mediated AmpC beta-lactamase genes in clinical isolates by using multiplex PCR. J. Clin. Microbiol. 40, 2153–2162 (2002). 12037080 10.1128/JCM.40.6.2153-2162.2002 PMC130804

[b24] Poirel L., Cabanne L., Vahaboglu H. & Nordmann P. Genetic environment and expression of the extended-spectrum beta-lactamase *bla*_PER-1_ gene in gram-negative bacteria. Antimicrob. Agents Chemother. 49, 1708–1713 (2005). 15855485 10.1128/AAC.49.5.1708-1713.2005 PMC1087670

[b25] Adams-Haduch J. M. *et al.* Genetic basis of multidrug resistance in *Acinetobacter baumannii* clinical isolates at a tertiary medical center in Pennsylvania. Antimicrob. Agents Chemother. 52, 3837–3843 (2008). 18725452 10.1128/AAC.00570-08 PMC2573138

[b26] Hu Q., Hu Z., Li J., Tian B. & Xu H. Detection of OXA-type carbapenemases and integrons among carbapenem-resistant *Acinetobactor baumannii in* a teaching hospital in China. J. Basic Microbiol. 51, 467–472 (2011). 21656808 10.1002/jobm.201000402

[b27] Bertini A. *et al.* Characterization and PCR-based replicon typing of resistance plasmids in *Acinetobacter baumannii*. Antimicrob. Agents Chemother. 54, 4168–4177 (2010). 20660691 10.1128/AAC.00542-10 PMC2944597

[b28] Poirel L., Naas T. & Nordmann P. Diversity, epidemiology, and genetics of class D beta-lactamases. Antimicrob. Agents Chemother. 54, 24–38 (2010). 19721065 10.1128/AAC.01512-08 PMC2798486

[b29] Toleman M. A., Spencer J., Jones L. & Walsh T. R. *bla*_NDM-1_ is a chimera likely constructed in *Acinetobacter baumannii*. Antimicrob. Agents Chemother 56, 2773–2776 (2012). 22314529 10.1128/AAC.06297-11 PMC3346620

[b30] Yoon E. J. *et al.* Origin in *Acinetobacter guillouiae* and dissemination of the aminoglycoside-modifying enzyme Aph(3')-VI. MBio 5, e01972–01914, 10.1128/mBio.01972-14 (2014). 25336457 PMC4212838

[b31] Zong Z. & Zhang X. *bla*_NDM-1_-carrying *Acinetobacter johnsonii* detected in hospital sewage. J. Antimicrob. Chemother. 68, 1007–1010 (2013). 23288403 10.1093/jac/dks505

[b32] Zhang W. J. *et al.* Complete sequence of the *bla*_NDM-1_-carrying plasmid pNDM-AB from *Acinetobacter baumannii* of food animal origin. J. Antimicrob. Chemother. 68, 1681–1682 (2013). 23449827 10.1093/jac/dkt066

[b33] Yang J. *et al.* Dissemination and characterization of NDM-1-producing *Acinetobacter pittii* in an intensive care unit in China. Clin. Microbiol. Infect. 18, E506–513 (2012). 23036089 10.1111/1469-0691.12035

[b34] Bogaerts P., Huang T. D., Rezende de Castro R., Bouchahrouf W. & Glupczynski Y. Could *Acinetobacter pittii* act as an NDM-1 reservoir for *Enterobacteriaceae*? J. Antimicrob. Chemother. 68, 2414–2415 (2013). 23732698 10.1093/jac/dkt201

[b35] Chen T. L. *et al.* Contribution of a plasmid-borne *bla*_OXA-58_ gene with its hybrid promoter provided by IS*1006* and an IS*Aba3*-like element to beta-lactam resistance in acinetobacter genomic species 13TU. Antimicrob. Agents Chemother. 54, 3107–3112 (2010). 20516281 10.1128/AAC.00128-10 PMC2916316

[b36] Poirel L., Mansour W., Bouallegue O. & Nordmann P. Carbapenem-resistant *Acinetobacter baumannii* isolates from Tunisia producing the OXA-58-like carbapenem-hydrolyzing oxacillinase OXA-97. Antimicrob. Agents Chemother. 52, 1613–1617 (2008). 18299404 10.1128/AAC.00978-07 PMC2346634

[b37] Marti S. *et al.* Characterization of the carbapenem-hydrolyzing oxacillinase OXA-58 in an *Acinetobacter* genospecies 3 clinical isolate. Antimicrob. Agents Chemother. 52, 2955–2958 (2008). 18505859 10.1128/AAC.00072-08 PMC2493133

[b38] Chen T. L., Wu R. C., Shaio M. F., Fung C. P. & Cho W. L. Acquisition of a plasmid-borne *bla*_OXA-58_ gene with an upstream IS*1008* insertion conferring a high level of carbapenem resistance to *Acinetobacter baumannii*. Antimicrob. Agents Chemother. 52, 2573–2580 (2008). 18443121 10.1128/AAC.00393-08 PMC2443897

[b39] Poirel L. & Nordmann P. Genetic structures at the origin of acquisition and expression of the carbapenem-hydrolyzing oxacillinase gene *bla*_OXA-58_ in *Acinetobacter baumannii*. Antimicrob. Agents Chemother. 50, 1442–1448 (2006). 16569863 10.1128/AAC.50.4.1442-1448.2006 PMC1426978

[b40] Fu Y. *et al.* Characterization of a novel plasmid type and various genetic contexts of *bla*_OXA-58_ in *Acinetobacter* spp. from multiple cities in China. PLoS One. 9, e84680, 10.1371/journal.pone.0084680 (2014). 24400107 PMC3882262

[b41] Evans B. A., Hamouda A., Towner K. J. & Amyes S. G. Novel genetic context of multiple *bla*_OXA-58_ genes in *Acinetobacter* genospecies 3. J. Antimicrob. Chemother. 65, 1586–1588 (2010). 20542900 10.1093/jac/dkq180

[b42] Ji S. *et al.* Prevalence of carbapenem-hydrolyzing class D beta-lactamase genes in *Acinetobacter* spp. isolates in China. Eur. J. Clin. Microbiol. Infect. Dis. 33, 989–997 (2013). 24374815 10.1007/s10096-013-2037-z

[b43] Chen T. L. *et al.* Comparison of one-tube multiplex PCR, automated ribotyping and intergenic spacer (ITS) sequencing for rapid identification of *Acinetobacter baumannii*. Clin. Microbiol. Infect. 13, 801–806 (2007). 17488329 10.1111/j.1469-0691.2007.01744.x

[b44] Chang H. C. *et al.* Species-level identification of isolates of the *Acinetobacter calcoaceticus-Acinetobacter baumannii* complex by sequence analysis of the 16S-23S rRNA gene spacer region. J. Antimicrob. Chemother. 59, 321–322 (2007). 15814977 10.1128/JCM.43.4.1632-1639.2005 PMC1081347

[b45] Ellington M. J., Kistler J., Livermore D. M. & Woodford N. Multiplex PCR for rapid detection of genes encoding acquired metallo-beta-lactamases. J. Antimicrob. Chemother. 59, 321–322 (2007). 17185300 10.1093/jac/dkl481

[b46] Kovach M. E. *et al.* Four new derivatives of the broad-host-range cloning vector pBBR1MCS, carrying different antibiotic-resistance cassettes. Gene 166, 175–176 (1995). 8529885 10.1016/0378-1119(95)00584-1

[b47] Haase J., Kalkum M. & Lanka E. TrbK, a small cytoplasmic membrane lipoprotein, functions in entry exclusion of the IncP alpha plasmid RP4. J. Bacteriol. 178, 6720–6729 (1996) 8955288 10.1128/jb.178.23.6720-6729.1996 PMC178567

[b48] Chansiripornchai N., Ramasoota P., Bangtrakulnonth A., Sasipreeyajan J. & Svenson S. B. Application of randomly amplified polymorphic DNA (RAPD) analysis for typing avian *Salmonella enterica subsp .enterica*. FEMS Immunol. Med. Microbiol. 29, 221–225 (2000). 11064269 10.1111/j.1574-695X.2000.tb01526.x

[b49] Clinical and Laboratory Standards Institute (CLSI). Performance Standards for Antimicrobial Susceptibility Testing; Twenty-Second Informational Supplement. CLSI document M100-S22, Vol. 32 (CLSI, 2012).

[b50] Andrews J. M. & Howe R. A. BSAC standardized disc susceptibility testing method (version 10). J. Antimicrob. Chemother. 66, 2726–2757 (2011). 21921076 10.1093/jac/dkr359

