# Supplementary material for: “Roar” of blaNDM-1 and “silence” of blaOXA-58 co-exist in Acinetobacter pittii
Source: Sci Rep. 2015 Mar 10;5:8976. doi: 10.1038/srep08976 (PMC5155454; doi:10.1038/srep08976)
Supplement: Supplementary Information — Supplementary data [file srep08976-s1.doc]

**“Roar” of *bla*_NDM-1_ and “silence” of *bla*_OXA-58_ co-exist in**

***Acinetobacter pittii***

Shuru Zhou,, Xin Chen,, Xiaobin Meng, Guoxiong Zhang,

Jie Wang, Dongsheng Zhou, and Xuemin Guo,,

**Table S1. Collection of *bla*_NDM-1_-containing genetic platforms**

| ***bla*_NDM-1_ gene cluster** | **Plasmid** | | | | | **Strain** | **Location** |
| --- | --- | --- | --- | --- | --- | --- | --- |
|  | **Name** | **Size (kb)** | | **Type** | |  |  |
| *apha6–*IS*Aba125–bla*_NDM-1_*–ble*_MBL_*–ΔtrpF–dsbC–tnpR–zeta* (this study) | pNDM-44551 | 41 | | novel (T4SS) | | *A.pittii* | GD, China |
| *apha6*–IS*Aba125–bla*_NDM-1_–*ble*_MBL_–*ΔtrpF–dsbC–cutA–ΔgroES–groEL–insE–*IS*Aba125–tnpR–zeta* [^1^](#_ENREF_1) | pNDM-BJ01 | 47.3 | | novel (T4SS) | | *A. Lwoffii* | BJ, China |
| *apha6*–IS*Aba125*–*bla*_NDM-1_–*ble*_MBL_–*ΔtrpF–dsbC–cutA–ΔgroES–groEL–insE–tnpR–zeta* [*^1^*](#_ENREF_1) | pNDM-BJ02 | 46.2 | | novel (T4SS) | | *A.Lwoffii* | BJ, China |
| *apha6–*IS*Aba125–bla*_NDM-1_*–ble*_MBL_*–ΔtrpF–dsbC–cutA–ΔgroES–groEL–insE–*IS*Aba125–tnpR–zeta* [^2^](#_ENREF_2) | pXBB1 | 47.3 | | novel (T4SS) | | *A.Johnsonii* | SC, China |
| *apha6–*IS*Aba125–bla*_NDM-1_*–ble*_MBL_*–ΔtrpF–tnpR–*IS*Aba16–zeta* [*^2^*](#_ENREF_2) | pXBC1 | 42.5 | | novel (T4SS) | | *A.Johnsonii* | SC, China |
| *apha6–*IS*Aba125–bla*_NDM-1_*–ble*_MBL_*–ΔtrpF–dsbC–cutA–ΔgroES–groEL*–*insE*–IS*Aba125* [^3^](#_ENREF_3) | pABC7926 | 30-50 | | unknown | | *A. haemolyticus* | HN, China |
| *apha6–*IS*Aba125–bla*_NDM-1_*–ble*_MBL_*–ΔtrpF–dsbC–cutA–ΔgroES–groEL–insE–Δpac–ISAba11–tnpR* [^4^](#_ENREF_4) | pAP-D499 | 45 | | novel (T4SS) | | *A. pittii* | BJ, China |
| *bla*_NDM-1_*–ble*_MBL_*–ΔtrpF–dsbC–cutA–ΔgroES–groEL* [^3^](#_ENREF_3) | pABZ78 | 30-50 | | unknown | | *A. lwoffii* | ZJ, China |
| *apha6–*IS*Aba125–bla*_NDM-1_*–ble*_MBL_*–ΔtrpF–dsbC–mph2–msr–tnpR–zeta* [*^5^*](#_ENREF_5) | pNDM-AB | 47.1 | | novel (T4SS) | | *A. baumannii* | GD, China |
| *apha6–*IS*Aba125–bla*_NDM-1_*–ble*_MBL_*–ΔtrpF* [*^6^*](#_ENREF_6) | pAL-1 | 270 | | unknown | | *A. lwoffii* | China |
| *apha6*–IS*26–*IS*Aba125–bla*_NDM-1_*–ble*_MBL_*–ΔtrpF–dsbC* [*^3^*](#_ENREF_3) | pABCA95 | 30-50 | | unknown | | *A. pittii* | AH, China |
| *Δorf–*IS*Aba125–bla*_NDM-1_*–ble*_MBL_*–ΔtrpF–tat–dct–*Δ*groES–groEL–*IS*CR21–Δpac–*IS*Aba125– Δorf*  [^7^](#_ENREF_7) | Located on chromosome | | | | | *A. baumannii* | Switzerland |
| *Δmfs–*IS*Aba125–bla*_NDM-1_*–ble*_MBL_*–ΔtrpF–ΔgroES–groEL–insE–*IS*Aba125–Δmfs* [^8^](#_ENREF_8) | Located on chromosome | | | | | *A. baumannii* | Germany |
| ΔIS*Aba125–*IS*5–*ΔIS*Aba125–bla*_NDM-1_*–ble*_MBL_*–trpF*Δ*–dsbC–cutA–groES*Δ*–groEL–insE–*IS*26–ΔygbJ* [^9^](#_ENREF_9) | pNDM-HN380 | | 54 | | IncX3 | *K. pneumoniae* | GD, China |
| IS*26–*ΔIS*Aba125–bla*_NDM-1_*–ble*_MBL_*–Δbla*_DHA_*–ampR* [^10^](#_ENREF_10) | pNDM-OM | | 87.2 | | IncL/M | *K. pneumoniae* | Oman |
| ΔIS*26–*ΔIS*Aba125–bla*_NDM-1_*–ble*_MBL_*–Δbla*_DHA_*–Δldh* [^11^](#_ENREF_11) | pKpANDM-1 | | 180 | | unknown | *K. pneumoniae* | ND, India |
| *tpnA–*ΔIS*Aba125–bla*_NDM-1_*–ble*_MBL_*–ΔtrpF–dsbC–ΔgroES–groEL–tnpA* [^12^](#_ENREF_12) | pNDM-MAR | | 267.2 | | IncH | *K. pneumoniae* | Morocco |
| *tnpA–*Δ*ISAba125–bla*_NDM-1_*–ble*_MB_*_L_–ΔtrpF–dsbC–ΔgroES–groEL–ΔtnpA–tnpR* [^13^](#_ENREF_13) | pKPX-1 | | 250 | | IncR/F | *K. pneumoniae* | ND, India |
| IS*Kpn14–*ΔIS*Aba125–bla*_NDM-1_*–ble*_MBL_*–*Δ*trpF–dsbC–cutA–ΔgroES–groEL* [^14^](#_ENREF_14) | pNDM-KN | | 162.7 | | IncA/C | *K. pneumoniae* | Kenya |
| IS*26–*ΔIS*Aba125–bla*_NDM-1_*–ble*_MBL_*–ΔtrpF–Δbla_DHA_–ampR* [^15^](#_ENREF_15) | pNDM-HK | | 88.8 | | IncL/M | *E. coli* | HK, China |
| *apha6–*IS*Aba125–bla*_NDM-1_*–ble*_MBL_*–ΔtrpF–dsbC–cutA–ΔgroES–groEL* (GenBank accession # JF714412) | pNDM102337 | | 166 | | Inc A/C | *E. coli* | Canada |
| *apha6–*IS*Aba1*–ΔIS*Aba125–bla*_NDM-1_*–ble*_MBL_*–ΔtrpF–dsbC–cutA–ΔgroES–groEL* (accession # JF503991) | pNDM10505 | | 166.7 | | Inc A/C | *E. coli* | Canada |
| IS*903–*ΔIS*Aba125–bla*_NDM-1_*–ble*_MBL_*–ΔtrpF–dsbC–cutA–ΔgroES–groEL–insE–*IS903 [^16^](#_ENREF_16) | pNDM-Dok01 | | 195.5 | | IncA/C | *E. coli* | Japan |
| IS*Ec33–*ΔIS*Aba125–bla*_NDM-1_*–ble*_MBL_*–ΔtrpF–*IS*Sen4* [*^17^*](#_ENREF_17) | p271A | | 45 | | IncN2 | *E. coli* | Bangladesh |
| IS*Aba125–bla*_NDM-1_*–ble*_MBL_*–ΔtrpF–Δbla*_DHA_*–ampR* [^18^](#_ENREF_18) | unnamed | | 300 | | IncHI1 | *E. coli* | Spain |

GD, Guangdong Province; BJ, Beijing; SC, Sichuan Province; HN, Hunan Province; ZJ, Zhejing; AH, Anhui; HK, HongKong; ND, New Delhi. The genetic surroundings of *bla*_NDM-1_ in pNDM-44551 were compared to those of other *bla*_NDM-1-_carrying plasmids, and a highly conserved region was observed, including a copy of incomplete or complete IS*Aba125* adjacent to the 5′-end *bla*_NDM-1_, *ble*_MBL_ and *ΔtrpF* adjacent to the 3′-end of *bla*_NDM-1_. This strongly suggests that *bla*_NDM-1_ genes from different strains have the same origin. However, *bla*_NDM-1_-carrying plasmids, which are epidemic in *Enterobacteriaceae* in China, are of other types, here represented by pNDM-HN380, a novel type of *bla*_NDM-1_-carrying incompatibility group X3 (IncX3) plasmids, recovered from six mainland Chinese patients hospitalized in Hong Kong.

**Table S2** Primers used in the study

| **Name** | **Nucleotide sequence (5′-3′)** | **Target gene, usage** | **Reference** |
| --- | --- | --- | --- |
| Ab-ITS F | CATTATCACGGTAATTAGTG | 16S-23S rRNA intergentic spacer, used for *A. baumannii* identification | [^19^](#_ENREF_19) |
| Ab-ITS R | AGAGCACTGTGCACTTAAG |
| RecA F | CCTGAATCTTCTGGTAAAAC | *RecA*, used for *Acinetobacter* spp. determination |
| RecA R | GTTTCTGGGCTGCCAAACATTAC |
| ABC-F | GTCGTAACAAGGTAGCCGTA | 16S-23S rRNA intergentic spacer, used for *A.calcoaceticus-*  *A. baumannii* Complex identification | [^20^](#_ENREF_20) |
| ABC-R | GGGTTYCCCCRTTCRGAAAT |
| OXA-23 F | ACTTGCTATGTGGTTGCTTC | *bla*_OXA-23_ | This study |
| OXA-23 R | TGGAAGCTGTGTATGTGCTA |
| OXA-24 F | GCACCTATGGTAATGCTCTTG | *bla*_OXA-24_ | This study |
| OXA-24 R | ACCAACCTACCTGTGGAGTA |
| OXA-58 F | CGATCAGAATGTTCAAGCGC | *bla*_OXA-_ | This study |
| OXA-58 R | AGAGCAATATCATCACCAGCT |
| NDM-1 F | CTTCCAACGGTTTGATCGTC | *bla*_NDM-1_ | [^21^](#_ENREF_21) |
| NDM-1 R | TAGTGCTCAGTGTCGGCATC |
| TEM F | CTTCCTGTTTTTGCTCACC | *bla*_TEM_ | [^22^](#_ENREF_22) |
| TEM R | AGCAATAAACCAGCCAGC |
| SHV F | ATTTGTCGCTTCTTTACTCGC | *bla*_SHV_ | [^23^](#_ENREF_23) |
| SHV R | TTTATGGCGTTACCTTTGACC |
| PER F | ATGAATGTCATTATAAAAGC | *bla*_PER_ | [^24^](#_ENREF_24) |
| PER R | AATTTGGGCTTAGGGCAGAA |
| CTX-M F | TTTGCGATGTGCAGTACCAGTAA | *bla*_CTX-M_ | [^23^](#_ENREF_23) |
| CTX-M R | CGATATCGTTGGTGGTGCCATA |
| CTX-M-2 F | AAATGTGCTGCTCCTTTCGTGAGC | *bla*_CTX-M-2_ | [^23^](#_ENREF_23) |
| CTX-M-2 R | AGGGTTCGTTGCAAGACAAGACTG |
| CMY-1 F | GCTGCTCAAGGAGCACAGGAT | *bla*_CMY-1_ | [^25^](#_ENREF_25) |
| CMY-1 R | CACATTGACATAGGTGTGGTGC |
| CMY-2 F | TGGCCAGAACTGACAGGCAAA | *bla*_CTX--2_ | [^25^](#_ENREF_25) |
| CMY-2 R | TTT CTCCTG AAC GTG GCT GGC |
| DHA F | AACTTTCACAGGTGTGCTGGGT | *bla*_DHA_ | [^25^](#_ENREF_25) |
| DHA R | CCGTACGCATACTGGCTTTGC |
| IMP-like F | CTACCGCAGCAGAGTCTTTG | *bla*_IMP-like_ | [^26^](#_ENREF_26) |
| IMP-like R | AACCAGTTTTGCCTTACCAT |
| VIM-like F | AGYGGTGAGTATCCGACAG | *bla*_VIM-like_ | This study |
| VIM-like R | ATGAAAGTGCGTGGAGAC |
| SIM-like F | TACAAGGGATTCGGCATCG | *bla*_SIM-like_ | [^27^](#_ENREF_27) |
| SIM-like R | TAATGGCCTGTTCCCATGTG |
| IS*Aba125* F | CGGGGTACCACGAGAGTGTTAGAACCCAT^a^ | IS*Aba125*-*bla*_NDM-1_-*Ble*_MBL_ | This study |
| IS*Aba125* R | CCGCTCGAGTTAGACTGTAGCTAAATCTCG |
| ISAba3 F | GCTCTAGAGTAAAACTTGAAGTGCGACA | IS*Aba3*-like-*bla*_OXA-58_-IS*Aba3* |
| BLE R | TCCCCCGGGTCAGTCGGGGTTCTGGATCA |
| AP2 | GTTTCGCTCC | Random amplified DNA fragments  Used for strain species differentiation by RAPD^b^ | [^28^](#_ENREF_28) |
| M13 | GACGGCCAGT | Random amplified DNA fragments  Used for strain species differentiation by RAPD | This study |
| *Aba*-16S rRNA F | GTAGCTTGCTACTGGACCTAG | *A. baumannii* 16S rRNA,used as qPCR internal control | [^29^](#_ENREF_29) |
| *Aba*-16S rRNA R | CATACTCTAGCTCACCAGTATCG |
| *E.coli*-16S rRNA F | CTCCTACGGGAGGCAGCAG | *E.coli* 16S rRNA,used as qPCR internal control | [^30^](#_ENREF_30) |
| *E.coli*-16S rRNA R | GWATTACCGCGGCKGCTG |
| N1F | TTGGAAGGATCGCGGCTGG | *bla*_NDM-1_DSR^c^, primer walking | This study |
| N2F | CGAACGCGTGGCCCAGTTG | *bla*_NDM-1_ DSR, primer walking | This study |
| N3F | AGCGAGGTGTCGTGGCACAG | *bla*_NDM-1_ DSR, primer walking | This study |
| N4F | CCATTGCAGGTATTCGGGCA | *bla*_NDM-1_ DSR, primer walking | This study |
| N5F | TGTAATCGCAGGCGATCTTC | *bla*_NDM-1_ DSR, primer walking | This study |
| N6F | AGGATTGCACATACTGGCAT | *bla*_NDM-1_ DSR, primer walking | This study |
| N7F | GCTGTGCCTGAAATTACATCA | *bla*_NDM-1_ DSR, primer walking | This study |
| N1R | CTTGTCCTGATGCGCGTGAG | *bla*_NDM-1_USR^d^, primer walking | This study |
| N2R | TAGGACGAGTATTCAGTGAC | *bla*_NDM-1_ USR, primer walking | This study |
| N3R | AAGCTCACGATAGATCGTACT | *bla*_NDM-1_ USR, primer walking | This study |
| N4R | CTCAGAGAGCCAACTCAACA | *bla*_NDM-1_ USR, primer walking | This study |
| N5R | GACGATTCAACAAATCACGC | *bla*_NDM-1_ USR, primer walking | This study |
| W1F | AGCTGGTGATGATATTGCTCT | *bla*_OXA-58_ DSR, primer walking | This study |
| W2F | GGACAGTTTCATCACTGCTT | *bla*_OXA-58_ DSR, primer walking | This study |
| W3F | GTTCGGCCTGCTGAATCAAT | *bla*_OXA-58_ DSR, primer walking | This study |
| W4F | CTATCTGACTGAGATACTCG | *bla*_OXA-58_ DSR, primer walking | This study |
| W1R | TGTGACAAACACAGCATCAGC | *bla*_OXA-58_ USR, primer walking | This study |
| W2R | CTCGTTTCGTATAACAGCCA | *bla*_OXA-58_ USR, primer walking | This study |

^a^ Restriction sites are underlined. ^b^ Random Amplified Polymorphic DNA analysis ^c^ DSR, downstream region; ^d^ USR, upstream region.

**
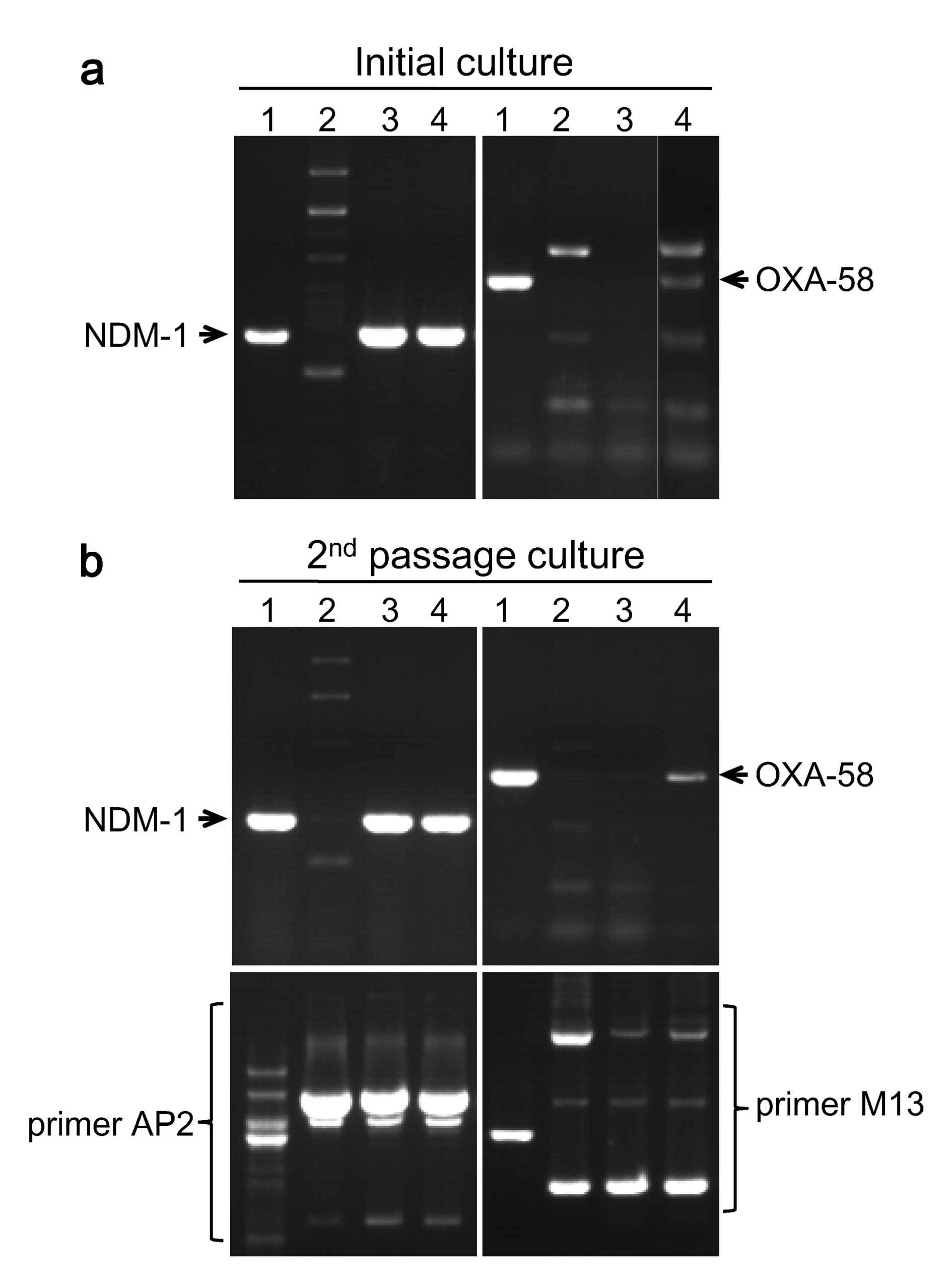
**

**Figure S1 | Detection of *bla*_NDM-1_ and *bla*_OXA-58_ by PCR.** Lane 1: genomic DNA of 44551; 2: genomic DNA of MZPB; 3: cell cultures of MZPB-44551; 4: cell cultures of MZPB-44551^OXA58+^. (a) The initial culture. A total of 50 MZPB conjugant colonies were picked randomly from the original selective plates and seeded into the liquid LB broth. These initial cultures were collected and boiled, and the respective supernatant was used as template for PCR detection of *bla*_NDM-1_ and *bla*_OXA-58_; all of them were positive for *bla*_NDM-1_, and five of them display showed weak PCR signal of *bla*_OXA-58_, which was further confirmed by sequencing. A *bla*_NDM-1_-positive and *bla*_OXA-58_-negative clone was designated MZPB-44551 and a *bla*_NDM-1_/*bla*_OXA-58_-positive clone designated MZPB-44551^OXA58+^. Shown are the PCR results for the initial cultures of MZPB-44551 and MZPB-44551^OXA58+^. (b) The second-passage culture. To exclude the false positive PCR signal due to donor DNA contamination, the initial culture of MZPB-44551 or MZPB-44551^OXA58+^ was spread onto the Amp^+^/PB^+^ plate, then one second-passage colony was randomly picked for each strain and subjected for PCR detection of *bla*_NDM-1_ and *bla*_OXA-58_ (upper panel).The strain species were differentiated by Random Amplified Polymorphic DNA with two short primers M13 and AP2 (lower panel). As expected, *bla*_OXA-58_ was detected by PCR in 44551 and MZPB-44551^OXA58+^ but not in MZPB and MZPB-44551.

**
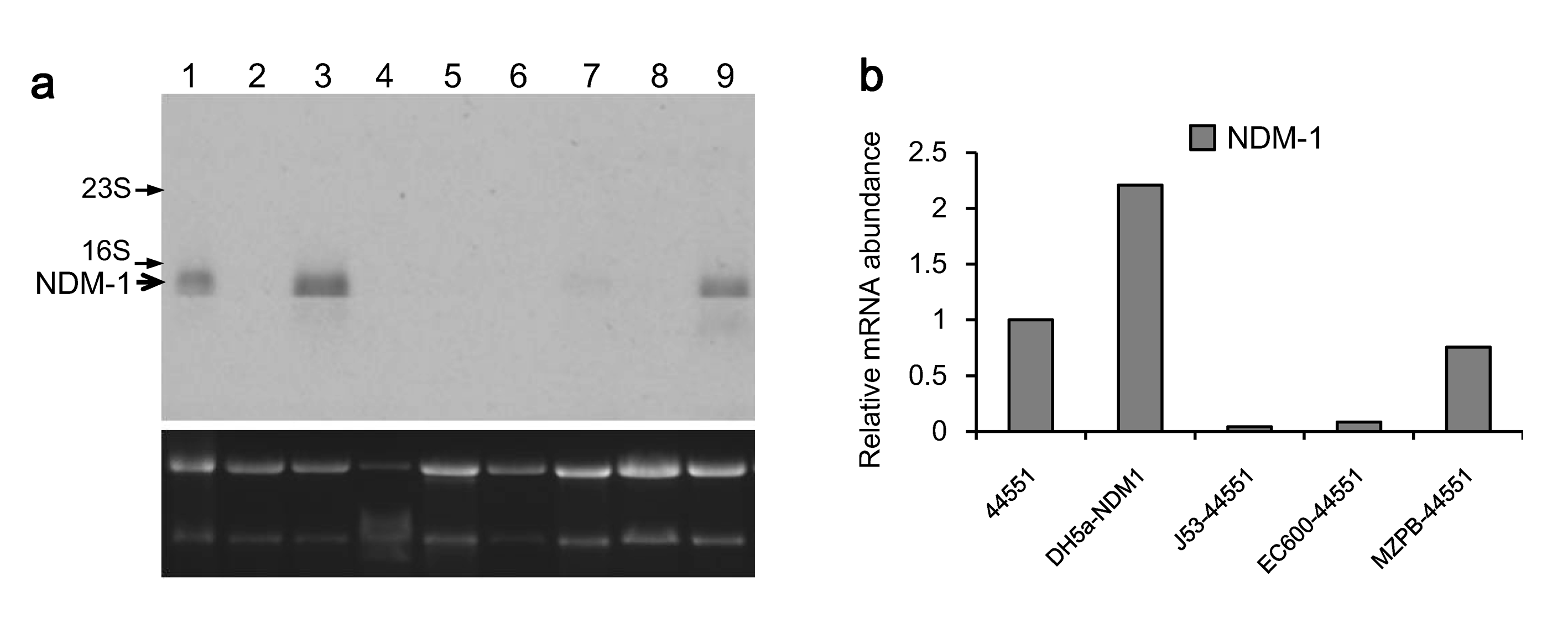
**

**Fig 4 Detection of *bla*_NDM-1_ transcripts.** (a) Northern blot. Total RNAs were extracted from strains 44551, DH5α, DH5α-NDM, J53, J53-44551, EC600, EC600-44551, MZPB, and MZPB-44551. RNA samples were analyzed on 1.2% agarose gel followed by EtBr staining, and then subjected to Northern blot hybridization with the DIG-labeled probe specific to blaNDM-1. Lane 1: 44551; 2: DH5α; 3: DH5α-NDM; 4: J53; 5: J53-44551; 6: EC600; 7: EC600-44551; 8: MZPB; 9: MZPB-44551. The EtBr staining of the 23S and 16S rRNA genes (2.9 kb and 1.5 kb, respectively) was used as loading control (lower panels). (b) RT-qPCR. The relative mRNA abundances of *bla*_NDM-1_ in 44551, DH5α-NDM, J53-44551, EC600-44551 and MZPB-44551 were detected by RT-qPCR. The 16S rRNA genes of 44551 and BL21 were employed as the internal control. The normalized mRNA abundance of *bla*_NDM-1_ in 44551 was set as 1.

**References**

1 Hu, H. *et al.* Novel plasmid and its variant harboring both a bla(NDM-1) gene and type IV secretion system in clinical isolates of Acinetobacter lwoffii. *Antimicrob Agents Chemother* **56**, 1698-1702, doi:10.1128/AAC.06199-11 (2012).

2 Zong, Z. & Zhang, X. blaNDM-1-carrying Acinetobacter johnsonii detected in hospital sewage. *J Antimicrob Chemother* **68**, 1007-1010 (2013).

3 Fu, Y. *et al.* Epidemiological characteristics and genetic structure of blaNDM-1 in non-baumannii Acinetobacter spp. in China. *J Antimicrob Chemother* **67**, 2114-2122 (2012).

4 Yang, J. *et al.* Dissemination and characterization of NDM-1-producing Acinetobacter pittii in an intensive care unit in China. *Clin Microbiol Infect* **18**, E506-513 (2012).

5 Zhang, W. J. *et al.* Complete sequence of the bla(NDM-1)-carrying plasmid pNDM-AB from Acinetobacter baumannii of food animal origin. *J Antimicrob Chemother* **68**, 1681-1682 (2013).

6 Wang, Y. *et al.* Identification of New Delhi metallo-beta-lactamase 1 in Acinetobacter lwoffii of food animal origin. *PLoS One* **7**, e37152 (2012).

7 Poirel, L. *et al.* Tn125-related acquisition of blaNDM-like genes in Acinetobacter baumannii. *Antimicrob Agents Chemother* **56**, 1087-1089 (2012).

8 Pfeifer, Y. *et al.* Molecular characterization of blaNDM-1 in an Acinetobacter baumannii strain isolated in Germany in 2007. *J Antimicrob Chemother* **66**, 1998-2001, (2011).

9 Ho, P.-L. *et al.* Identification and characterization of a novel incompatibility group X3 plasmid carrying blaNDM-1 in Enterobacteriaceae isolates with epidemiological links to multiple geographical areas in China. *Emerging Microbes & Infections* **1**, e39 (2012).

10 Poirel, L., Al Maskari, Z., Al Rashdi, F., Bernabeu, S. & Nordmann, P. NDM-1-producing Klebsiella pneumoniae isolated in the Sultanate of Oman. *J Antimicrob Chemother* **66**, 304-306 (2011).

11 Yong, D. *et al.* Characterization of a new metallo-beta-lactamase gene, bla(NDM-1), and a novel erythromycin esterase gene carried on a unique genetic structure in Klebsiella pneumoniae sequence type 14 from India. *Antimicrob Agents Chemother* **53**, 5046-5054 (2009).

12 Villa, L., Poirel, L., Nordmann, P., Carta, C. & Carattoli, A. Complete sequencing of an IncH plasmid carrying the blaNDM-1, blaCTX-M-15 and qnrB1 genes. *J Antimicrob Chemother* **67**, 1645-1650 (2012).

13 Huang, T. W. *et al.* Copy Number Change of the NDM-1 sequence in a multidrug-resistant Klebsiella pneumoniae clinical isolate. *PLoS One* **8**, e62774 (2013).

14 Carattoli, A., Villa, L., Poirel, L., Bonnin, R. A. & Nordmann, P. Evolution of IncA/C blaCMY-(2)-carrying plasmids by acquisition of the blaNDM-(1) carbapenemase gene. *Antimicrob Agents Chemother* **56**, 783-786 (2012).

15 Ho, P. L. *et al.* Complete sequencing of pNDM-HK encoding NDM-1 carbapenemase from a multidrug-resistant Escherichia coli strain isolated in Hong Kong. *PLoS One* **6**, e17989 (2011).

16 Sekizuka, T. *et al.* Complete sequencing of the bla(NDM-1)-positive IncA/C plasmid from Escherichia coli ST38 isolate suggests a possible origin from plant pathogens. *PLoS One* **6**, e25334 (2011).

17 Poirel, L., Lagrutta, E., Taylor, P., Pham, J. & Nordmann, P. Emergence of metallo-beta-lactamase NDM-1-producing multidrug-resistant Escherichia coli in Australia. *Antimicrob Agents Chemother* **54**, 4914-4916 (2010).

18 Sole, M. *et al.* First description of an Escherichia coli strain producing NDM-1 carbapenemase in Spain. *Antimicrob Agents Chemother* **55**, 4402-4404 (2011).

19 Chen, T. L. *et al.* Comparison of one-tube multiplex PCR, automated ribotyping and intergenic spacer (ITS) sequencing for rapid identification of Acinetobacter baumannii. *Clin Microbiol Infect* **13**, 801-806 (2007).

20 Chang, H. C. *et al.* Species-level identification of isolates of the Acinetobacter calcoaceticus-Acinetobacter baumannii complex by sequence analysis of the 16S-23S rRNA gene spacer region. *J Clin Microbiol* **43**, 1632-1639 (2005).

21 Islam, M. A. *et al.* Emergence of multidrug-resistant NDM-1-producing Gram-negative bacteria in Bangladesh. *Eur J Clin Microbiol Infect Dis* **31**, 2593-2600 (2012).

22 Feizabadi, M. M. *et al.* Distribution of bla(TEM), bla(SHV), bla(CTX-M) genes among clinical isolates of Klebsiella pneumoniae at Labbafinejad Hospital, Tehran, Iran. *Microb Drug Resist* **16**, 49-53 (2010).

23 Adams-Haduch, J. M. *et al.* Genetic basis of multidrug resistance in Acinetobacter baumannii clinical isolates at a tertiary medical center in Pennsylvania. *Antimicrob Agents Chemother* **52**, 3837-3843 (2008).

24 Poirel, L., Cabanne, L., Vahaboglu, H. & Nordmann, P. Genetic environment and expression of the extended-spectrum beta-lactamase blaPER-1 gene in gram-negative bacteria. *Antimicrob Agents Chemother* **49**, 1708-1713 (2005).

25 Perez-Perez, F. J. & Hanson, N. D. Detection of plasmid-mediated AmpC beta-lactamase genes in clinical isolates by using multiplex PCR. *J Clin Microbiol* **40**, 2153-2162 (2002).

26 Takeyama, K. *et al.* Multidrug-resistant Pseudomonas aeruginosa isolated from the urine of patients with urinary tract infection. *J Infect Chemother* **8**, 59-63 (2002).

27 Ellington, M. J., Kistler, J., Livermore, D. M. & Woodford, N. Multiplex PCR for rapid detection of genes encoding acquired metallo-beta-lactamases. *J Antimicrob Chemother* **59**, 321-322 (2007).

28 Chansiripornchai, N., Ramasoota, P., Bangtrakulnonth, A., Sasipreeyajan, J. & Svenson, S. B. Application of randomly amplified polymorphic DNA (RAPD) analysis for typing avian Salmonella enterica subsp. enterica. *FEMS Immunol Med Microbiol* **29**, 221-225 (2000).

29 Kuo, H. Y., Chang, K. C., Kuo, J. W., Yueh, H. W. & Liou, M. L. Imipenem: a potent inducer of multidrug resistance in Acinetobacter baumannii. *Int J Antimicrob Agents* **39**, 33-38 (2012).

30 Stackebrandt, E. & Goodfellow, M. *Nucleic acid techniques in bacterial systematics*. (Wiley, 1991).
